# Saphenous veins in coronary artery bypass grafting need external support

**DOI:** 10.1177/0218492320980936

**Published:** 2020-12-13

**Authors:** Ninos Samano, Domingos Souza, Michael R Dashwood

**Affiliations:** 1 Department of Cardiothoracic and Vascular Surgery and University Health Care Research Center, Faculty of Medicine and Health, Örebro University, Örebro, Sweden; 2 Surgical and Interventional Sciences, Royal Free Hospital Campus, University College Medical School, London, UK

**Keywords:** Coronary artery bypass, saphenous vein, stents, tissue and organ harvesting, vascular patency

## Abstract

The saphenous vein is the most commonly used conduit for coronary artery bypass grafting. Arterial grafts are harvested with the outer pedicle intact whereas saphenous veins are harvested with the pedicle removed in the conventional graft harvesting technique. This conventional procedure causes considerable vascular damage. One strategy to improve vein graft patency has been to provide external support. Ongoing studies show that fitting a metal external support improves conventionally harvested saphenous vein graft patency. On the other hand, the no-touch technique of harvesting the saphenous vein provides an improved graft with long-term patency comparable to that of the internal mammary artery. This improvement is suggested to be due to preservation of vessel structures. Interestingly, many of the mechanisms proposed to be associated with the beneficial actions of an artificial external support on saphenous vein graft patency are similar to those underlying the beneficial effect of no-touch saphenous vein grafts where the intact outer layer acts as a natural support. Additional actions of external supports have been advocated, including promotion of angiogenesis, increased production of vascular-protective factors, and protection of endothelial cells. Using no-touch harvesting, normal vascular architecture is maintained, tissue and cell damage is minimized, and factors beneficial for graft patency are preserved. In this review, the significance of external support of saphenous vein grafts in coronary artery bypass grafting is discussed.

## Conduits in coronary artery bypass grafting

The saphenous vein (SV) was first introduced as a conduit for coronary revascularization five decades ago.^1^ It remains the most commonly used conduit for coronary artery bypass grafting (CABG) in over 90% of procedures worldwide.^2^ The SV is the conduit of choice because its superficial position renders it easily accessible, it is expendable because deeper vessels maintain blood flow after its removal, and its abundant length allows for multiple grafts. Furthermore, the SV in humans differs from other veins that generally remain under fairly constant flow and pressure, because the SVs of the lower limbs are subjected to variable orthostatic pressures caused by alterations in posture and movement.^3^ Apart from structural differences compared to other veins,^4^ being subjected to these pressure variations may precondition segments of SV grafts when subjected to the hemodynamics of the coronary arterial system.

**Figure 1.**
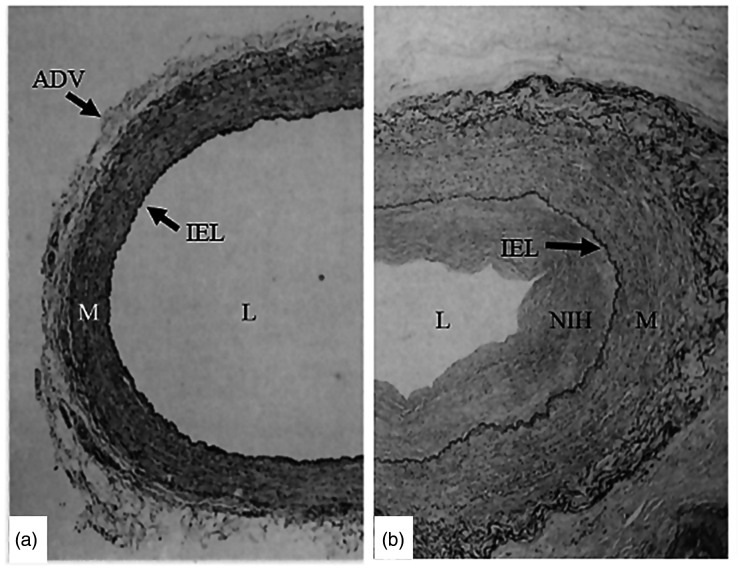
Histology of conventional saphenous veins at harvest and six months after coronary artery bypass grafting. (a) At harvest the lumen (L) is dilated due to high-pressure distension and the adventitia (ADV) has been partially removed/damaged. (b) Six months after coronary artery bypass grafting, a substantial degree of neointimal hyperplasia (NIH) is seen in the graft at the luminal side of the internal elastic lamina (IEL). The media (M) and neoadventitia of the graft are thicker than at harvesting. Modified from Cox et al. Stranger in a strange land - the pathogenesis of saphenous-vein graft stenosis with emphasis on structural and functional differences between veins and arteries. *Prog Cardiovasc Dis* 1991;34:45–68.

**Figure 2.**
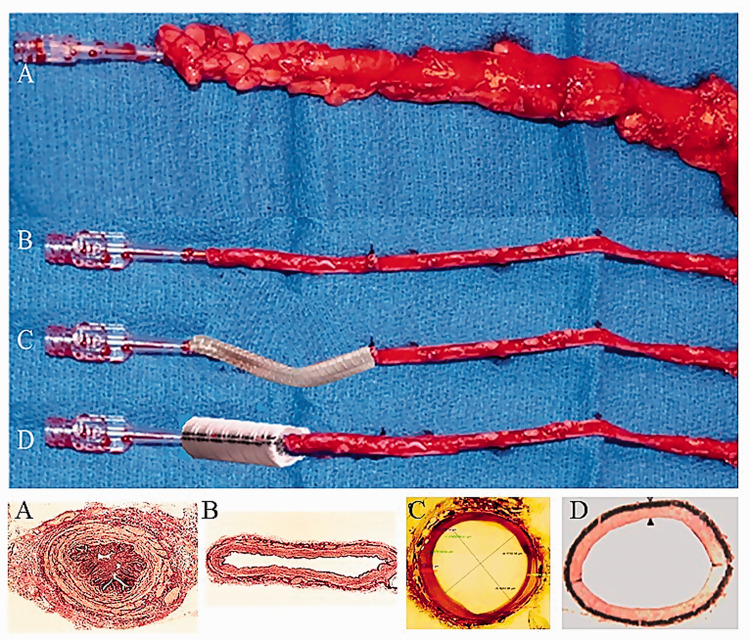
Explanted saphenous veins as bypass grafts. Top panel: (A) a no-touch saphenous vein harvested with the surrounding cushion of fat intact, not distended, and with minimal damage to the vein. (B) Conventional saphenous vein that has been distended and with the cushion of fat removed and the adventitia damaged (similar to saphenous vein at harvesting in Figure 1). (C) Conventional saphenous vein with a braided cobalt external stent fitted. Superimposed stent as described in Ben-Gal et al. Expandable external support device to improve saphenous vein graft patency after CABG. *J Cardiothorac Surg* 2013;8:122. (D) Conventional saphenous vein with a superimposed Dacron external stent fitted. Reproduced from Jeremy et al. On the biology of saphenous vein grafts fitted with external synthetic sheaths and stents. *Biomaterials* 2007;28:895–908. Lower panel: Transverse histological sections. (A) No-touch saphenous vein with adventitia intact, which has not been distended. (B) Conventionally prepared saphenous vein with the surrounding cushion of fat removed, adventitia damaged, and distended with saline at 300 mm Hg pressure. A and B from Dashwood et al. Retaining perivascular tissue of human saphenous vein grafts protects against surgical and distension-induced damage and preserves endothelial nitric oxide synthase and nitric oxide synthase activity. *J Thorac Cardiovasc Surg *2009;138:334–40. (C) Sheep saphenous vein after fitting an external cobalt stent, in which adventitial damage has occurred. Perfusion fixed at 100 mm Hg. Reproduced from Ben-Gal et al. Expandable external support device to improve saphenous vein graft patency after CABG. *J Cardiothorac Surg* 2013;8:122. (D) Control un-grafted pig saphenous vein with damaged adventitia. Perfusion fixed at 100 mm Hg. Reproduced from Jeremy et al. On the biology of saphenous vein grafts fitted with external synthetic sheaths and stents. *Biomaterials* 2007;28:895–908.

Use of the left internal mammary artery (LIMA) and radial artery has been encouraged because of their good long-term patency, long-term survival, and freedom from reinterventions,^5^,^6^ compared to the high incidence of early graft occlusion, progressive intimal hyperplasia, and late graft atherosclerosis of conventional (CT) SV grafts (Figure 1).^7^ Although extensive arterial revascularization in CABG is strongly promoted, the benefits of these conduits may be short-lived or not determined in certain cases.^8^ Consequently, the SV remains the most common and an indispensable conduit in CABG, and its long-term patency is one of the most crucial challenges in cardiovascular surgery.^9^ Paradoxically, while both LIMA and radial artery grafts are often harvested with their outer pedicle intact, according to the original instructions of Favaloro,^1^ the SV pedicle should be removed (Figure 2). This traumatic form of harvesting causes considerable vascular damage to the SV, damage that potentially affects all vessel layers and may partially account for the inferior patency of the SV compared to the LIMA.^9^,^10^ Moreover, skeletonization of the internal mammary artery is also performed in some centers and reported to be associated with comparable patency to pedicled internal mammary artery.^11^ With this in mind, it seems reasonable to say that the surrounding tissue is more important in venous rather than arterial grafts.

## Improving saphenous vein graft patency using external support

The potential use of external supports in improving vein graft performance was first proposed over 50 years ago.^12^ Here, placement of a monofilament knitted tube increased the patency of jugular vein grafts compared to controls in a dog bypass model. Fifteen years later, using the same experimental model, placement of an external Dacron stent was shown to reduce intimal hyperplasia and increased the density of the vasa vasorum in the vein graft wall.^13^ Both processes played an important beneficial role in vein graft performance. Although other forms of support have been described, including external mesh^14^ and fibrin glue,^14^,^15^ it is the external stents that have received most attention recently. A pig model of arteriovenous bypass grafting was developed and used extensively to study the potential effect of external stent placement on SV graft patency (Figure 2).^16^,^17^ This group showed that using the pig SV graft model, application of an external polyester stent to the outside of carotid interposition SV grafts reduced intimal hyperplasia and total wall thickness one month after implantation. In addition, morphological changes and platelet-derived growth factor expression in stented grafts and contralateral nonstented grafts in the same pigs were studied six months after graft implantation. Here, reduced medial thickening, neointima formation, and cell proliferation were sustained in the externally stented grafts, effects that were associated with a significant reduction in platelet-derived growth factor expression.^18^ While these observations support the use of artificial external stents as a means of improving SV graft performance, the potential for the various stent materials used to stimulate an inflammatory response needs to be considered. Such a scenario might be anticipated, particularly at the stent/SV interface, triggering various mechanisms underlying the progression of atherosclerosis. Indeed, this has been described in the pig bypass model where inflammatory cells, in particular macrophages and giant cells, were shown to infiltrate the external stent. In addition, certain prosthetic materials used, such as Vicryl sheaths and polyglactin stents, stimulate the release of inflammatory and immune cells, releasing factors that influence vascular smooth muscle cell proliferation and migration, neointimal hyperplasia (NIH) formation and angiogenesis.^17^ Furthermore, increases in prostacyclin I2, cyclic adenosine monophosphate, and cyclic guanosine monophosphate formation were noted in both stented and nonstented grafts compared to ungrafted SV, with the production being greater in the stented compared to the nonstented graft. Also, in the adventitia of stented vein grafts, nitric oxide synthase was associated with microvessels as well as inflammatory cells. Taken together, these data are suggestive of a role for prostacyclin I2 and nitric oxide in promoting micro-angiogenesis in the adventitia of stented vein grafts, which may in turn minimize graft hypoxia, an established contributory factor to neointima formation. While an inflammatory response to external supports has been demonstrated in the pig and other animal bypass models, as far as we are aware, this has not been studied in sections of human SV grafts where external support has been used.

Since there were concerns regarding potential long-term complications of a permanent polyester stent, a subsequent study was undertaken. This study investigated the effect of a biodegradable external stent on vein graft thickening at one month and the longer-term effects at six months. The loose-fitting biodegradable polyglactin external stent reduced porcine vein graft thickening at one month and this persisted in the long-term, even after degradation of the stent itself. The authors concluded that biodegradable external stents have potential advantages over permanent stent material in clinical application.^19^ The results of a small randomized trial of the Extent device (a macroporous Dacron sheath reinforced with polytetrafluoroethylene ribs) were disappointing.^20^ Here, 20 patients received an Extent-supported graft to either the right or the left coronary system. However, in all 17 patients returning for follow-up, the Extent-supported grafts were found to be thrombosed on angiographic assessment (6 and 19 months postoperatively). On the other hand, all of the LIMA grafts and non-stented SV grafts remained patent. The authors attributed this to a combination of stent rigidity, oversizing, and incomplete tube design leading to graft kinking either at the anastomoses or in the middle of the graft. Since potential problems associated with stent rigidity and testing of biodegradable stents in the pig model had been recognized earlier, it seems surprising that Dacron stents were used in CABG patients. These bench-to-bedside/translational studies failed, most likely due to various limitations of the pig experimental model that was used.^10^

More recently, an external support made from braided cobalt-chromium-nickel-molybdenum-iron alloy fibres, forming an expandable external SV support, has been developed and tested in a sheep bypass model (Figure 2).^21^ Here, 14 sheep underwent CABG with one SV graft supported and a non-supported SV serving as a control. Early and late quantitative angiography was performed with histological evaluation carried out on graft sections on completion of the study. Ten animals survived with 3 of the control grafts thrombosed and one occluded. Although there was no difference in uniformity between grafts immediately after CABG, there was significant (*p* < 0.002) nonuniformity in the control versus supported grafts. This was confirmed by histological evaluation showing that the mean intimal area of the supported grafts was significantly lower than that in the control grafts (*p* < 0.02). The authors admit the limitations of their study, in particular the small sample size and short follow-up period. Also, the quality of the histological sections provided in the figures casts doubt on the accuracy of the morphometric data. Furthermore, ongoing venous external support trials (VEST) are in progress on patients undergoing CABG. Despite the positive data from VEST,^22^,^23^ the patient numbers are low and the follow-up period short. In a recently published study, Taggart and colleagues^23^ reported the results of VEST IV with comparable vein graft failure rates between stented and nonstented grafts (30% and 23% respectively, *p* = 0.42) at a mean time 4.5 years. On the other hand, intimal hyperplasia and the development of luminal irregularities were significantly reduced by external stenting.

## No-touch saphenous vein graft harvesting

The studies above described various means of providing external support to SVs that have been conventionally harvested by stripping the veins of their surrounding tissue. When preparing the SV in this way, the vessel is subjected to surgical trauma resulting in vascular damage. This is recognized to affect graft patency and perhaps partially explains the inferior SV graft performance compared to the LIMA.^7^,^10^ In contrast to CT harvesting, the no-touch (NT) technique of SV harvesting introduced by Souza^24^ preserves the cushion of surrounding tissue (Figure 2). The course of the SV is marked preoperatively on the leg using Doppler ultrasound. This enables preoperative assessment of vein quality and size and performing an incision directly over the SV, thus minimizing the risk for unnecessarily long incisions and tissue flaps. The SV is then harvested using diathermy and scissors to include a surrounding fat pedicle of approximately 0.5 cm. Avoiding direct contact with the vessel prevents spasm and the need for manual distension.^25^ Side-branches are ligated and divided at the same distance from the SV. After removal, the SV is stored in heparinized blood. When harvested in this fashion, the SV is subjected to minimal trauma and retains its normal architecture,^10^,^26^ resulting in graft patency comparable to that of the LIMA at up to 16 years of follow-up (Table 1).^27^,^28^ Crude SV graft patency was 64% in CT vs. 83% in NT, *p* = 0.03, which is similar to the patency of the LIMA, 88% at 16 years. In addition, an intravascular ultrasound series showed a 4-fold increase of grafts with considerable intimal hyperplasia and a 2-fold increase in grafts with atherosclerotic plaques in CT compared to NT SV grafts at up to 8.5 years follow-up.^29^

**Table 1.** Ratio of the number of patent grafts to total number of grafts for the two surgical techniques with follow-up after 1.5, 8.5, and 16 years.*

	Conventional			No-touch			Group difference in % patency^†^		
Follow-up (years)	1.5	8.5	16	1.5	8.5	16	1.5	8.5	16
No. of patients	46	37	27	45	37	27			
Single grafts	96/107 (90%)	68/87 (78%)	41/63 (65%)	103/109 (94%)	78/87 (90%)	55/67 (82%)	0.23	0.05	0.06
Sequential grafts	16/20 (80%)	10/14 (71%)	5/9 (56%)	15/15 (100%)	14/14 (100%)	7/8 (87%)	0.12	0.10	0.29
All grafts	112/127 (89%)	78/101 (77%)	46/72 (64%)	118/124 (95%)	92/101 (91%)	62/75 (83%)	0.08	0.01	0.03

*Reproduced from Samano et al. The no-touch saphenous vein for coronary artery bypass grafting maintains a patency, after 16 years, comparable to the left internal thoracic artery: a randomized trial. *J Thorac Cardiovasc Surg* 2015;150:880–8. ^†^Tested with multilevel logistic regression except for sequential grafts where Fisher’s exact test had to be used due to small numbers and cells with no occluded grafts.

## Comparison of synthetic and natural external stents; mechanical properties

Prior to grafting, a typical vein will be subjected to low pressures (∼5–8 mm Hg), nonpulsatile flow, and a shear stress of ∼0.2 dyne·cm^−2^. However, supine and orthostatic SV pressures in healthy volunteers have been reported to be 7 ± 1 and 76 ± 2 mm Hg, respectively.^30^ Subsequent to grafting in the arterial system, the SV will be subjected to high pressures (∼60–140 mm Hg), pulsatile flow, and shear stress of ∼3–6 dyne·cm^−2^.^31^ Within a month, these conditions result in SV graft remodelling and the development of diffuse intimal hyperplasia. Over time, intimal thickening and atherosclerotic lesions are formed specifically at regions where disturbed flow occurs (Figure 1).^32^ Vein graft thickening is markedly asymmetric producing clear alterations of blood flow through the graft. Turbulent blood flow subsequently elicits thrombosis and hyperplasia.^33^,^34^ A major feature of external stents or sheaths is that they impose symmetry on the graft when it thickens in response to arterial hemodynamics. This in turn, imposes laminar and symmetric blood flow, thereby reducing hyperplasia.^17^

**Figure 3.**
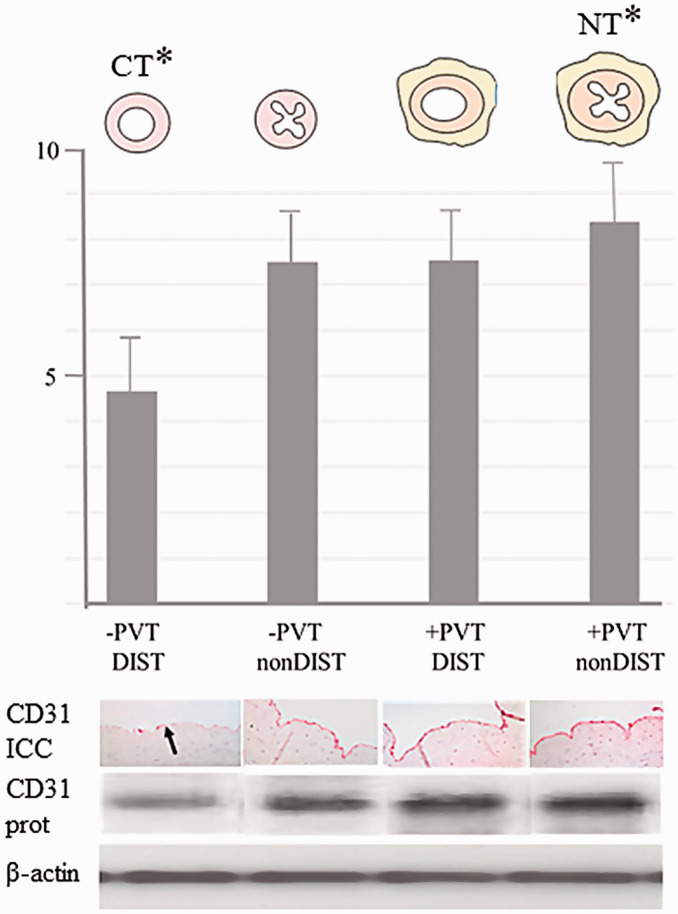
Perivascular tissue protects against distension-induced endothelial damage. Top panel: line drawings of saphenous veins harvested by the conventional technique (CT) in which perivascular tissue has been removed and distension used (-PVT DIST); saphenous vein section with perivascular tissue removed but not distended (-PVT nonDIST); saphenous vein with perivascular tissue intact and distended (+PVT DIST) and no-touch (NT) saphenous vein section with perivascular tissue intact and not distended (+PVT nonDIST). The longitudinal axis represents the density of Western blots in arbitrary units. Lower panel: a histogram showing quantitative measurement (mean of 8 Western blots) for CD31 protein levels, as an indicator of endothelial cell integrity. Immunohistochemistry showing representative CD31 immunostaining in which there is reduced endothelial staining (the arrow shows endothelial denudation with a few intact endothelial cells) in CT saphenous vein compared to all other preparations where the endothelium is virtually intact. CD31 protein shows representative CD31 Western blots for all 4 preparations of SV. β-actin = protein control. Modified from Dashwood et al. Retaining perivascular tissue of human saphenous vein grafts protects against surgical and distension-induced damage and preserves endothelial nitric oxide synthase and nitric oxide synthase activity. *J Thorac Cardiovasc Surg *2009;138:334–40.

**Figure 4.**
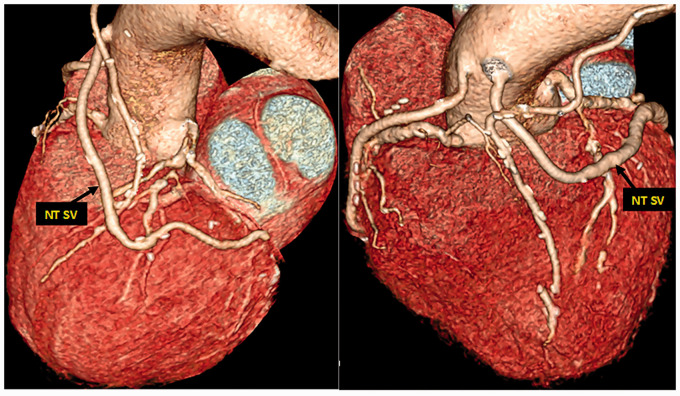
Computed tomography angiography eight years postoperatively, showing a sequential no-touch (NT) saphenous vein (SV) graft anastomosed to two obtuse marginal branches. There is less risk of kinking despite the excess length and shifting course of the SV graft.

The perivascular fat also plays an important, indirect role in protecting the luminal endothelium from the deleterious consequences of damaging this important single layer of cells. When using CT SV harvesting, a high percentage of SVs go into spasm that is overcome using high-pressure manual distension. Such pressures have been shown to be as high as 560 mm Hg,^35^ potentially even reaching 2000 mm Hg.^36^ These pressures cause serious endothelial damage and exposure of the intimal basement membrane. With the NT technique, the SV is handled by its outermost cushion and as there is no direct contact with the SV by surgical instruments, spasm rarely occurs. Hence, there is no need for distension and endothelial damage is minimized.^25^,^26^,^37–39^ An additional beneficial effect of preserved outer layers of the SV is the protection conferred on the luminal endothelium once inserted as a graft and subjected to arterial hemodynamics. A study comparing sections of CT versus NT harvested SVs showed marked differences in endothelial integrity.^26^ Here, certain SV segments were subjected ex vivo to intraluminal saline distension at 300 mm Hg pressure, well within the range of manual pressure used in CT SV harvesting.^40^ While there was marked endothelial damage in CT SVs, the endothelium of NT SVs that were not distended was virtually intact. An additional two treatments were performed in this study where CT SVs (i.e., segments stripped of the outer layer) were not distended and NT SVs (segments with the cushion of surrounding tissue intact) were distended. The endothelium of SVs in both groups remained relatively undamaged (Figure 3).^26^ There are three conclusions to be drawn from these results: it is mainly the distension, not the surgery, that causes endothelial damage to CT SVs; the cushion of surrounding tissue acts as a buffer and protects the endothelium against high-pressure distension of NT SVs; if the cushion of surrounding tissue in NT SVs protects against 300 mm Hg distension at harvesting, it will protect the endothelium once implanted as a graft where it is subjected to systolic arterial pressure usually ranging from 100 to 180 mm Hg.^10^,^26^ Another mechanical aspect of external support is protecting long vein grafts from kinking (Figure 4). This feature is provided with both artificial and natural external support, with one important distinction when using sequential grafts. No-touch SV grafts are easily anastomosed to more than one target coronary artery, whereas fitting artificial external stents to sequential grafts is a technical challenge with many pitfalls.

Certainly, high-pressure distension at harvest and altered turbulence/shear both stimulate various mechanisms of graft failure, particularly those associated with NIH and thrombus formation. This may account, at least in part, for the detrimental effects described in the Extent trial where all Extent SV grafts were thrombosed but non-stented SV and internal mammary artery grafts remained patent.^20^ In earlier pig studies, end-to-end SV-to-carotid artery grafting was performed, a criticism raised in relation to grafts used in CABG.^41^ Subsequently, the more recent pig studies have used end-to-side grafts. Altered hemodynamics were investigated in a small study comparing stented vs. nonstented SV grafts in which resin casts were used to assess changes in graft geometry and the potential effect on blood flow.^42^ At 1-month post-implantation, unstented graft geometry was irregular, with nonuniform dilatation, substantial axial lengthening, curvature, kinking, and possible long-pitch helical distortion. In contrast, stented grafts showed a reduction in these changes although fine corrugation, occasional slight kinking or narrowing of segments, and possible long-pitch helical distortion were observed in some instances. In a later review, the effect of external stents or sheaths on the symmetry of grafts in response to arterial hemodynamics were discussed. Here, vein graft thickening was described as markedly asymmetric, a condition causing marked alterations in turbulent blood flow, which can elicit thrombosis and NIH. External stents or sheaths were said to keep the graft contained in a way that prevents turbulent blood flow and imposes laminar and symmetric blood flow, thereby reducing NIH.^17^ While these studies were performed using the pig vein graft model, more recent hemodynamic studies in patients have been described using VEST. A method for analyzing and comparing SV grafts following CABG was used to study flow dynamics inside vein grafts with and without support using VEST. Specific computational fluid dynamic measurements were used to characterize the relevant hemodynamic parameters and flow conditions in a small number of patients in whom stented and nonstented SV grafts were assessed at 12 months post-transplantation. SV grafts surrounded by VEST were associated with similar time-averaged wall shear stress, yet had fewer lesions, a lower oscillatory shear index and relative residence time index, and more uniform flow with fewer flow discrepancies. Based on these data, the authors concluded that VEST improved hemodynamic factors that correlate with graft failure following CABG.^22^

## Perivascular fat

Interestingly, early beneficial aspects of the pig external stent model were proposed in a review based on a conference presentation where issues regarding stent size, porosity, and fit were discussed.^18^ In the questions and answers section, Professor Wallwork asked: “do you not think that one of the issues about looking at a chronic ejection model is that you strip the outer layer of the vessel, therefore you are making it ischemic? Would you not get the same effect if you took the vein out with a lot of fat around it instead of stripping it? It would be a nice experiment to see whether that would give you the same results as putting the stent around it.”^43^ This would indeed have been “a nice experiment”. If acted upon, over 30 years ago, supporting evidence may have shown that preserving the perivascular fat contributes to improved performance of SV grafts as subsequently described in SVs harvested by the NT technique.^10^,^27^,^28^

It is now recognized that perivascular fat is not merely a means of mechanical support for blood vessels, and there has been much interest in the beneficial effects of so called adipocyte-derived relaxing factors; for a review, see Gollasch and Dubrovska.^44^ Of particular relevance to conduits used for CABG are results from in-vitro studies showing that perivascular adipose tissue has an anticontractile action on both the internal mammary artery and SV.^45^,^46^ The potential role of perivascular fat-derived factors in SV graft performance is discussed in detail in a number of recently published research studies and reviews.^47^,^48^

## Adventitial microvessels

There is experimental evidence from a variety of animal models for a role of the adventitial vasa vasorum in maintaining a healthy vessel. For example, rapid development of atherosclerotic lesions in rabbit carotid artery is induced by perivascular placement of a close-fitting collar. This resulted in NIH caused by obstruction of the adventitial vasa vasorum and creation of mural ischemia.^49^ Furthermore, removal of the adventitia of the carotid artery induced NIH, but intimal hyperplasia regression was observed on the appearance of a neoadventitia associated with the restoration of medial oxygenation.^50^ Interestingly, in tissue-engineered blood vessels developed as potential substitutes for autologous bypass grafts, the appearance of “adventitial” vasa vasorum has been described after implantation of the engineered graft.^51^

**Figure 5.**
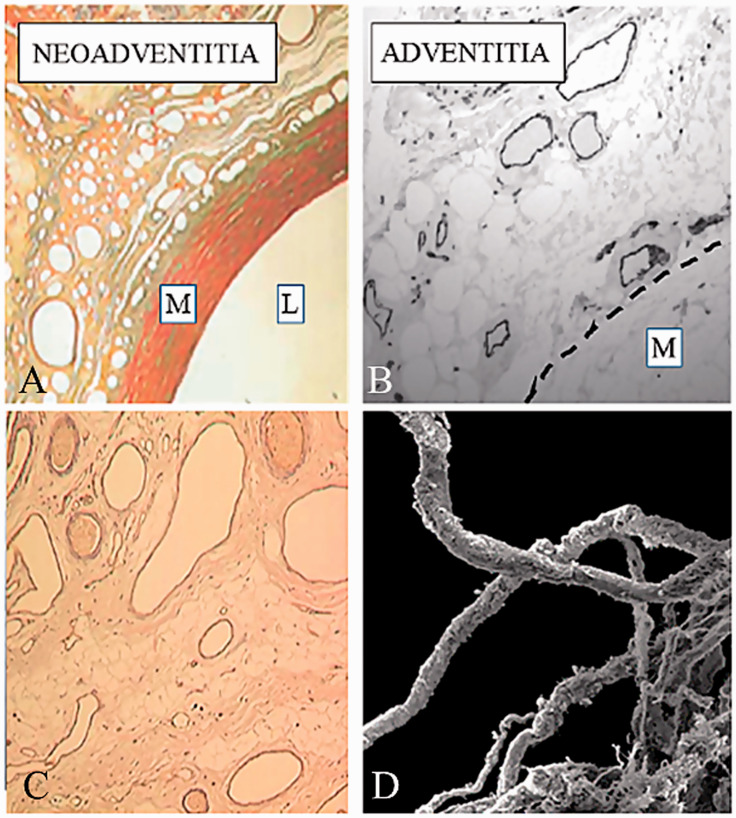
Adventitial microvessels: a role in graft patency. (A) Microvessels/neovascularization (neoadventitia) at the Extent-outer vessel interface 6 months after implantation. L: lumen; M: media. Reproduced from Jeremy et al. On the biology of saphenous vein grafts fitted with external synthetic sheaths and stents. *Biomaterials* 2007;28:895–908. (B) Adventitial vasa vasorum in a no-touch saphenous vein (SV) graft (endothelial cells identified by CD34 immunostaining). The interrupted line indicates the media-adventitia border. The vasa vasorum and associated endothelial nitric oxide synthase is more important for saphenous vein than arterial bypass grafts. Reproduced from Dreifaldt et al. The vasa vasorum and associated endothelial nitric oxide synthase is more important for saphenous vein than arterial bypass grafts. *Angiology* 2013;64:293–9. (C) Immunostaining of microvessels in the neo-adventitial region of an Extent stented vein graft one month after implantation, which indicates endothelial cells lining microvessels and active angiogenesis. Reproduced from Jeremy et al. On the biology of saphenous vein grafts fitted with external synthetic sheaths and stents. *Biomaterials* 2007;28:895–908. (D) Scanning electron micrograph of damaged vasa vasorum at the adventitia-media border of a conventional saphenous vein. Reproduced from Vasilakis et al. Human saphenous vein and coronary bypass surgery: scanning electron microscopy of conventional and ‘no-touch’ vein grafts. *Vasc Dis Prevent* 2004;1:133–9.

Regarding the suggested mechanisms underlying the pig vein graft/external stent model, an exudate was observed in the gap between the stent and the graft within a week. This exudate was rich in fibrin and fibrinogen which are both potent angiogenic factors. Over the following weeks, microvessels appeared in the space between the graft and the stent, which proceeded to organize into a well-defined structure, much of which was comprised of microvessels. The formation of “neo-vasa vasorum” within the exudate in the graft-stent interface is suggested to play an important beneficial role in the pig external stent model.^17^ Based on these observations, it has been proposed that, by means of angiogenesis, the regeneration of the vasa vasorum may constitute an important adaptation of vein grafts to arterial conditions and to the repair and reintegration of the microvascular system.^17^ The question arises, why promote angiogenesis in damaged SVs artificially when, in patients undergoing CABG, the SV vasa vasorum remains intact when harvested by the NT technique? The vasa vasorum is a microvessel network providing the adventitia and media with oxygen and nutrients and is more pronounced and penetrates deeper towards the lumen in veins than in arteries. When harvesting the SV conventionally, the adventitia and associated structures are often damaged, including damage to the vasa vasorum. Scanning electron microscope studies have identified various aspects of damage to the vasa vasorum, including severing of these microvessels at the adventitia-media border (Figure 5).^52^ In addition, ultrastructural studies have shown constriction and collapse of numerous vasa vasorum microvessels and clumping of erythrocytes.^53–55^ Such features would ultimately reduce transmural blood flow, potentially leading to NIH and thus affecting graft patency.^49^,^50^ By contrast, the vasa vasorum of NT SV grafts remains intact with normal endothelial and vascular smooth muscle cell morphology.^53^ An unexpected finding in this study was the observation of small openings within the luminal endothelium, suggested to represent terminations of the vasa vasorum that extend from the adventitial-medial network.^52^,^54–56^ These luminal terminations may play an important role in the improved performance of NT SV grafts because filling of the adventitial vasa vasorum has been observed in such grafts perfused during CABG.^39^,^54^ This has also been confirmed ex vivo in isolated segments of NT SV grafts perfused with blood from the cardiopulmonary bypass machine.^39^,^54^ In a recent ex-vivo study using India ink perfusion of SVs from CABG patients, sections from these veins showed staining of the luminal endothelium and the vasa vasorum throughout the vessel wall, extending to the capillary network within the perivascular fat.^57^

As oxygenated blood passes through the lumen of arteries, the endothelium and vessel wall receive sufficient oxygen and nutrients by diffusion. However, the vasa vasorum in veins represents a microvessel network with the principle role of supplying blood to the wall as luminal oxygen and nutrient levels are low. Hence, the vasa vasorum of veins penetrate closer towards the intima than those of arteries, and are seen to advantage in the thick walls of the SV.^4^ The potential importance of the vasa vasorum in SV grafts has been investigated, comparing their regional distribution and density.^54^ The density, size, and total area of the vasa vasorum in the media and adventitia of transverse histological sections was assessed by computer-assisted morphometry comparing NT with CT SV grafts. Although there was no significant difference between density and size in these vessel layers, there was a significantly greater total area in both the media and adventitia of NT vs. CT SVs.^54^ A subsequent study revealed striking differences between SV and arterial grafts. The density of the vasa vasorum was not only far higher in SV vs. arterial grafts but this study provided quantitative support for the previously described penetration of vasa vasorum in veins vs. arteries.^4^ The vasa vasorum was virtually absent in the media and significantly lower in the adventitia of both the LIMA and radial artery compared to the SV.^58^ Also, of particular relevance, is the significant reduction in the total number of vasa vasorum when stripping the adventitia, as occurs in CT SV harvesting.^54^ Therefore, in CT grafts, there is a high risk of developing hypoxic or ischemic conditions in the SV wall and initiating detrimental processes that affect graft patency.^55^

## Concluding remarks

We agree with the concept and strongly advocate that SV grafts will perform better if equipped with external support. However, it is surprising that while recognizing the damage caused to the SV when harvesting conventionally, various strategies that are potentially harmful, complicated, time-consuming, or costly are pursued. Nevertheless, using the relatively simple surgical technique of NT harvesting preserves normal SV architecture, providing a superior graft for patients undergoing CABG. The recent success of artificial external stents for the SV in CABG provides yet another argument for harvesting this vein with its natural stent of perivascular fat. Ongoing and future studies will determine whether the benefit of synthetic external stents will translate into superior SV graft patency over the longer term. The discrepancy between how most arterial and venous grafts are harvested has been challenged since the development of the NT SV graft harvesting technique. The technique was recommended to be used in open harvesting of the SV by the European Society of Cardiology and the European Association for Cardiothoracic Surgery in 2018. We unpretentiously leave the decision to our surgical colleagues to determine which technique is most suitable for each individual patient, with a pertinent quotation from Benjamin Franklin: “An ounce of prevention is worth a pound of cure”.^59^
